# Special Issue “Epigenetics in Neurodegenerative Diseases”

**DOI:** 10.3390/ijms25073647

**Published:** 2024-03-25

**Authors:** Simone Agostini, Roberta Mancuso

**Affiliations:** IRCCS Fondazione Don Carlo Gnocchi, 20148 Milan, Italy; sagostini@dongnocchi.it

Epigenetic mechanisms inducing phenotypic changes without altering the DNA genome are increasingly recognized as key factors modulating gene expression and, consequently, cell functions. In particular, all these modifications are even involved in brain development, differentiation and maturation, with a great impact on human neurodegenerative diseases.

Neurodegenerative diseases, such as Multiple Sclerosis (MS), Alzheimer’s Disease (AD), Parkinson’s Disease (PD) and Amyotrophic Lateral Sclerosis (ALS), are complex multifactorial pathologies; although the molecular mechanisms at the basis of their pathogenesis remain obscure, genes and the environment are important contributing factors to their development. It is now clear that gene–environment interactions are mediated by epigenetic changes, which may represent the mechanistic bridge between these two elements, and for this reason they have been increasingly implicated in the development of different neurodegenerative diseases. The brain’s adaptations to environmental stressors, inflammation and injury involve the transcriptional and post-transcriptional epigenetic mechanisms, aspects about which little is still known but that can provide a better understanding of the etiopathogenesis of these pathologies and open new roads for promising pharmacological and clinical therapies.

The present Special Issue focused on different epigenetic mechanisms that, by altering DNA structure through DNA methylation, histone modification or modulating gene expression by non-coding RNAs, such as microRNAs (miRNAs) or long non-coding-RNAs (lncRNAs), are involved in several processes associated with neurodegenerative diseases, such as the accumulation of misfolded proteins in the central nervous system, neuroinflammation and microglia activation, oxidative stress, apoptosis, autophagia and the formation and maturation of synapses (synaptic plasticity).

The possible epigenetic elements shared among prion diseases and prion-like neurodegenerative diseases have been analyzed in an interesting review [1]: the accumulation of misfolded proteins in the central nervous system (CNS) and their spreading in different brain regions is a common mechanism observed both in transmissible spongiform encephalopathies (TSEs of humans or animals) and in Amyotrophic Lateral Sclerosis and Alzheimer’s, Parkinson’s and Huntington’s diseases (HD). Amyloid beta (Aβ), tau, α-synuclein and prion (Pr^c^) proteins share similar pathogenetic properties [2], although their infectious nature has only been demonstrated for Pr^c^ [3]. Consequently, the study of animal and cellular models of prion disease may provide a useful model from which to better understand the regulatory role of epigenetic mechanisms on misfolded proteins.

Increasing evidence shows epigenetic modifications in all these pathologies, but the mechanisms seem to act differently in each of these diseases; in the scientific literature, several authors have reported different trends in global methylation and acetylation profiles, altered levels of histone acetyltransferase (HAT) or histone deacetylases (HDACs) enzymes, and many miRNA profiles in prion-like neurodegenerative diseases. These findings may allow the identification of useful biomarkers and new targets for therapies, although it is clear that we are only just beginning to understand the complex regulatory mechanisms and molecular pathways contributing to the pathogenesis of neurodegenerative diseases.

Another aspect focused on in this Special Issue is the regulatory interplay between different epigenetic mechanisms (such as methylation, acetylation and non-coding RNA expression), an emerging field of RNA epigenetics. In particular, it is known that a group of miRNAs, known as epi-miRNAs, can modulate key mediators of the epigenetic machinery, such as DNA methyltransferases (DNMTs) or HDACs; moreover, they can also be regulated by the same epigenetic mechanism: hypermethylation of a large number of promoters of miRNAs has been evidenced in multiple tumors [4], resulting in alterations in the expression of genes involved in cancers and disruptions of the physiological processes implicated in carcinogenesis. Only a few studies have focused on other diseases, such as cardiovascular [5] or autoimmune [6] diseases.

One interesting article published in this Special Issue studied the mechanisms regulating microRNA expression in MS [7]. 

This paper focused on hydroxymethylation in the regulation of three microRNAs (miR-155-5p, miR-326 and miR-223-3p) critically involved in the regulation of inflammatory response and neuroinflammation [8]; these microRNAs have been found to be deregulated not only in autoimmune [9] but also in neurodegenerative diseases [10]. The paper of Basak et al. reported an increased level of modifications of promotors for the three miRNAs, and in particular a correlation between hydroxymethylation and the level of miR-223-3p expression in lymphocytes of MS patients compared to controls.

This study also contributes to highlighting the mechanism of epigenetic miRNA regulation in neurological diseases; further studies will be necessary to gain a broader picture of how these complex reciprocal interactions among epigenetic mechanisms may have a role in the development of neurodegenerative diseases, for the purpose of suggesting new therapeutic approaches.

Importantly, for all these neurodegenerative diseases, physical activity and rehabilitation programs are recommended for their beneficial effects that may help to slow down clinical progression or at least to mitigate clinical symptoms. Several studies showed that physical exercise influences epigenetic mechanisms [11], both in healthy subjects and in subjects with neurodegenerative diseases [12,13,14]; therefore, it is plausible that exercise-induced beneficial effects may involve a modulation of epigenetic processes to restore physiological functions. Future studies will be important to identify epigenetic biomarkers useful for supporting these non-pharmacological measures to prevent the evolution of neurodegenerative disease, as well as to predict the outcome and the efficiency of a particular rehabilitation program.
